# Comparison of sensory outcomes in patients with successful motor outcome versus recurrent exotropia after surgery for intermittent exotropia

**DOI:** 10.1038/s41598-022-17067-5

**Published:** 2022-08-01

**Authors:** Hye Jun Joo, Jin Ju Choi, Jin Woo Ro, Dong Gyu Choi

**Affiliations:** 1 Department of Ophthalmology, Hallym University College of Medicine, Seoul, Korea; 2 Moon’s Eye Clinic, Suwon, Korea; 3 Kangnam the Light Eye Centre, Seoul, Korea

**Keywords:** Diseases, Eye diseases

## Abstract

Here, we compared sensory outcomes between patients with successful motor outcomes and recurrent exotropia after intermittent exotropia surgery. We retrospectively analyzed 303 patients who underwent surgery for intermittent exotropia, divided into two groups: successful motor outcome defined as an alignment between 10 prism diopters (PD) exodeviation and 5PD esodeviation at the final follow-up (Group A, n = 177) and residual or recurrent exotropia defined as exodeviation > 10 PD (Group B, n = 126). Preoperative and postoperative (at final visit) sensory outcomes were compared using the Titmus stereotest and distance Worth 4-dot test. Stereoacuity significantly improved postoperatively in both successful motor outcome group (Group A) and residual or recurrent exotropia group (Group B). However, stereoacuity did not differ between groups preoperatively and postoperatively. On the other hand, fusion rates for the Worth 4-dot test were significantly higher in group A than in group B, preoperatively and postoperatively, and significantly increased postoperatively only in group A. Therefore, the distance Worth 4-dot test may be useful for evaluating postoperative prognosis and preoperative sensory status.

## Introduction

Stereopsis is generated by the fusion of horizontally disparate retinal images and is considered the highest standard of binocular vision^1^. Stereoacuity provides a measure of the quality of binocular vision, thereby becoming a useful objective measure of control and severity in intermittent exotropia [X(T)]^2–4^.

Deterioration in distance stereoacuity has been considered as a good measurement for control of severity in X(T) and used as a means to determine the need for surgery. Surgical correction of exodeviation leads to significant improvement in distance stereoacuity^5–10^. However, there are controversies surrounding the effects of near stereoacuity in X(T). Several previous studies demonstrated that most patients with X(T) have normal near stereoacuity until later stages of the disease^11–13^. However, some studies showed that patients with X(T) exhibit reduced near stereoacuity^14^. Similarly, some studies have suggested surgical realignment to improve near stereoacuity^15^, while others have suggested that near stereoacuity test is minimally affected by surgery^5,16–19^.

In contrast, Yildirim et al. reported that diminished distance stereoacuity may not be the most sensitive indicator of X(T) control; rather, they suggested that central suppression test should be used to assess sensory status in patients with X(T)^20^. Similarly, Epstein and Tredici reported that central suppression occurs before the loss of distance stereoacuity in X(T)^21^. They showed that some patients with microexostrabismus demonstrated a monocular suppression scotoma in the binocular visual field on the distance vectographic alternate-letter suppression test; however, these patients retained distance stereoacuity on the vectographic contour circles test.

This study compared the sensory outcomes using near Titmus stereotest and distance Worth 4-dot test between patients with successful motor outcome and those with recurrent exotropia following surgery for intermittent exotropia.

## Methods

The medical records of 303 patients (147 males, 156 females) who had undergone surgery for intermittent exotropia with poor control between September 1999 and August 2018 and a postoperative follow-up period of 6 months or more were retrospectively reviewed.

The exclusion criteria were as follows: (1) previous surgery for exotropia, (2) sensory exotropia occurring from unilateral visual impairment, (3) coexistent restrictive or paralytic strabismus, (4) patients with follow-up periods shorter than 6 months postoperatively, (5) low cooperation in the Titmus stereotest, (6) neurologic disorders, and (7) consecutive esotropia (defined as esodeviation of ≥ 10 prism diopters [PD] at one month postoperatively or later). Patients who showed reliable Titmus stereotest results, but low cooperation in the Worth 4 dot test, were also included in this study. All surgeries were performed by the same surgeon (D.G.C.).

The control of exodeviation was scaled as good, fair, or poor. Good control was defined as fusion breaks only after cover testing at distance fixation that resumed rapidly without the need for blinking or refixation. Fair control was defined as blinking or refixation to control deviation after disruption with cover testing at distance fixation. Poor control was defined as breaking spontaneously without any form of fusion disruption or not spontaneously regaining alignment despite blinking or refixation^22^.

The study protocol adhered to the Declaration of Helsinki and was approved by the institutional review board of Hallym University Medical Centre (2021–12-018) who waived the requirement for informed consent due to the retrospective nature of the study.

### Grouping

The patients were divided into two groups according to the surgical outcomes: those with successful motor outcome, which was defined as an alignment between 10 PD of exodeviation and 5 PD of esodeviation at the final follow-up (Group A), and those with recurrent exotropia defined as exodeviation > 10 PD (Group B).

### Preoperative ophthalmologic examination

All patients underwent complete ophthalmological examinations, including cycloplegic refraction with 1% cyclopentolate chloride (Cyclogyl, Alcon Lab. Inc., Fort Worth, TX, USA), and 1% tropicamide (Mydriacyl, Alcon Lab. Inc.). The angle of deviation was determined by the prism and alternate cover test at distance and near (6 m and 33 cm, respectively) in all fields of gaze using accommodative targets with the best optical corrections. If the exodeviation at distance was larger than 10 PD compared with that at near distance, either eye of the participants was patched for 1 h to eliminate fusional convergence^23^.

### Measurement of sensory status

Titmus stereotest (Stereo Optical Co., Chicago, IL, USA) comprises three portions (fly, animal, circle) and can estimate down to 40 arcsec. The test was performed at near distance of 40 cm while wearing polarized glasses. When in doubt whether the patient actually had stereoscopic vision, we occluded one eye and inquired whether there was a difference in appearance. Additionally, because only horizontal disparity produces stereopsis, we turned the plate at 90°, to block out the stereoscopic effect. The Titmus stereotest was performed under normal illumination, and there was no time limit for the response. For the analysis, the results of the Titmus stereotest were graded into three categories based on the degree of stereopsis: good (40–60 arcsec), moderate (80–200 arcsec), and poor (> 200 arcsec).

The Worth 4-dot test was performed at 6 m under dark conditions with the participants wearing red-green glasses over their own glasses. The results of the Worth 4-dot test were as follows: (1) fusion, if four lights were seen, (2) suppression, if two or three lights were seen, and (3) diplopia, if five lights were seen. Tests were performed at least twice to reduce test variability.

In this study, the results of the Titmus stereotest and Worth 4-dot test performed on the day of the visit just before the surgery and the measurements at the final follow-up were used as pre- and post-operative sensory status data, respectively, for the analysis.

### Surgery

All surgeries were performed under general anesthesia by the same surgeon (D.G.C.) according to the modified formula from the surgical table suggested by Parks (based on the angle of distant deviation) (Table 1)^24^. All patients underwent conventional strabismus surgery consisting of bilateral lateral rectus recession (BLR) or unilateral recess-resect (R&R) and unilateral lateral rectus recession (ULR) in the non-dominant eye. Either BLR or R&R were selected by the operating surgeon, who had no preference for BLR or R&R. ULR could be performed in cases of exotropia of < 25 PD.

**Table 1:** Surgical table based on the angle of distant deviation.

Deviation at distance, PD	Unilateral LR recession(mm)	Bilateral LR recession (mm)	R&R (mm)
15	8	4.0	4.0/3.0
20	9	5.0	5.0/4.0
25	10	6.0	6.0/5.0
30	–	7.0	7.0/5.5
35	–	7.5	7.5/6.0
40	–	8.0	8.0/6.5
50	–	9.0	9.0/7/0

*PD* prism dioptres, *LR* lateral rectus, *R and R* unilateral lateral rectus recession and medical rectus resection.

### Postoperative management

Postoperative alignment was measured on postoperative day 1, months 1, 3, and 6, and at the final follow-up. Alternate full-time patching was prescribed for patients who complained of diplopia or developed esodeviation postoperatively and was continued until diplopia or esodeviation disappeared. If the esodeviation persisted for 2 months after the operation, cycloplegic refraction was performed, and refractive errors were re-corrected. If the esotropia did not disappear with alternate patching for 2 months, base-out Fresnel press-on prisms (3 M Press-On Optics; 3 M Health Care, St Paul, MN, USA) were prescribed until the esotropia resolved^23^.

### Outcome measures

The main outcome was the differences in preoperative and postoperative results of the Titmus stereotest and Worth 4-dot tests in groups A and B, respectively. Comparison of the sensory status between groups A and B using the Titmus stereotest and Worth 4-dot test was also performed, preoperatively and postoperatively.

### Statistical analysis

The Chi-square test, Fisher’s exact test, and Independent T-test were used for the analysis. Statistical analyses were conducted using SPSS software for Windows, Version 26.0 (IBM Corp., Armonk, NY, USA). Statistical significance was set at p < 0.05.

### Ethics approval and informed consent

The study protocol adhered to the Declaration of Helsinki and was approved by the institutional review board of Hallym University Medical Centre (2021-12-018) who waived the requirement for informed consent due to the retrospective nature of the study.

## Results

Table 2 shows the demographic data of the patients in group A (successful motor outcome group, n = 177) and Group B (residual or recurrent exotropia group, n = 126). The preoperative mean exodeviation was 24.62 ± 7.23 PD in Group A and 26.69 ± 6.76 in group B at distance (Independent T-test, p = 0.01), 23.51 ± 9.57 and 25.31 ± 9.17 at near (p = 0.103). The mean age at surgery was 8.9 ± 5.5 (range, 2.6 − 41) years in group A and 7.2 ± 3.5 (2.3–30.3) in group B (p = 0.001). The mean postoperative follow-up period was 42.0 ± 36.2 months (range, 6–196) in group A and 45.5 ± 39.6 (range, 6–257) months in group B (p = 0.320). There were no differences between the groups based on sex, X(T) classification, surgery type, and accompanying strabismus (p > 0.05).

**Table 2:** Demographic data of the patients in groups A and B.

Variables	Group A (n = 177)	Group B (n = 126)	p-value
Sex (male:female)	82:95	65:61	0.815 *
Age at surgery (years)	8.9 ± 5.5	7.16 ± 3.46	0.001 ^†^
**X(T) classification**			0.629 *
Basic	87.57% (155/177)	90.48% (114/126)	
Divergence excess	1.13% (2/177)	0.79% (1/126)	
Pseudodivergence excess	10.17% (18/177)	8.73% (11/126)	
Convergence insufficiency	1.13% (2/177)	0% (0/126)	
**Preoperative angle of exodeviation (PD)**			
At distance	24.50 ± 7.45	26.69 ± 6.76	0.01 ^†^
At near	23.51 ± 9.57	25.31 ± 9.17	0.103 ^†^
**Associated strabismus**			
Vertical deviation^c^	25 (14.1%)	17 (13.6%)	0.897 *
Oblique muscle dysfunction	25 (14.1%)	27 (21.4%)	0.097 *
Dissociated vertical deviation	4(2.3%)	1(0.8%)	0.308 ^‡^
**Surgical method**			0.242 *
Unilateral LR recession	27.7% (49/177)	19.8% (25/126)	
Bilateral LR recession	9.0% (16/177)	7.9% (10/126)	
R&R	63.3% (112/177)	44.8% (91/126)	
Postoperative follow-up period (months)	42.0 ± 36.2	45.5 ± 39.6	0.320 ^†^

Group A = alignment between 10 PD exodeviation and 5 PD esodeviation at the last follow-up; Group B = residual or recurrent exotropia defined as exodeviation > 10 PD at last follow-up; X(T) = intermittent exotropia; PD = prism dioptres; LR = lateral rectus; R&R = unilateral lateral rectus recession and medical rectus resection.

* Chi-square test.

^†^ Independent T-test.

^‡^ Fisher’s exact test.

^†^p-value.

**Figure 1:**
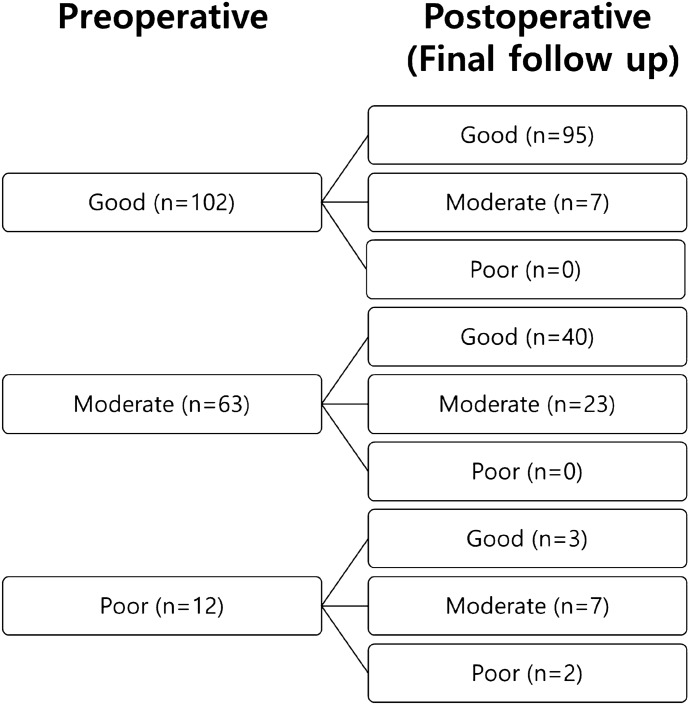
Postoperative changes in the Titmus stereotest results grouped by the preoperative stereoacuity results in Group A.

**Figure 2:**
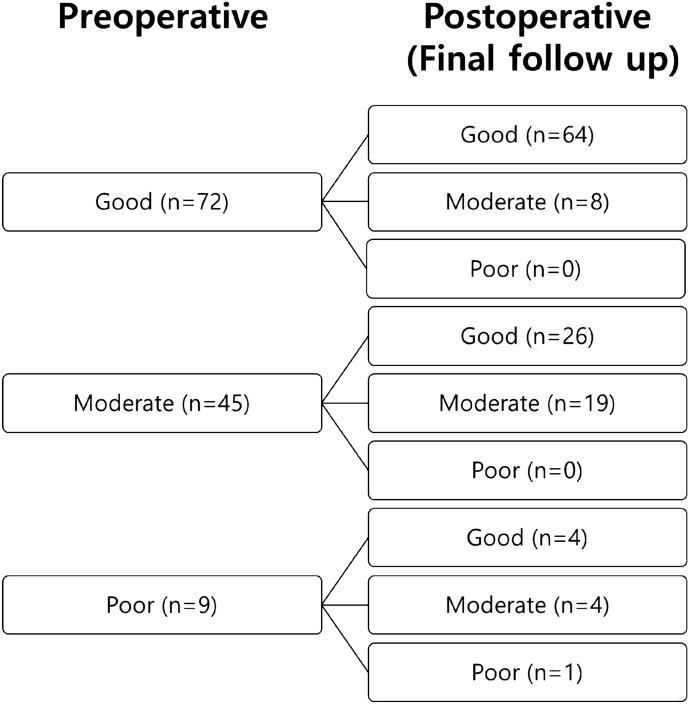
Postoperative changes in the Titmus stereotest results grouped by preoperative stereoacuity results in Group B.

In the Titmus stereotest, good, moderate, and poor stereopsis was observed in 102 (57.6%), 63 (35.6%), and 12 (6.8%) patients preoperatively in group A, respectively, and 72 (57.1%), 45 (35.7%), and 9 (7.1%) patients in group B, respectively, which showed no significant statistical difference between the groups (chi-square test, p = 0.991). At the final visit, good, moderate, and poor stereopsis were achieved in 138 (78%), 37 (20.9%), and 2 (1.1%) of group A, and in 94 (74.6%), 31 (24.6%), and 1 (0.8%) of group B, respectively, which showed no significant statistical difference between the groups (p = 0.726). However, stereopsis significantly improved after surgery compared to the preoperative results in both groups A and B (p < 0.001) (Table 3). In detail, in group A, patients with poor preoperative near stereoacuity showed improvement in 83% (good 25%, moderate 58%) and remained the same in 17%, postoperatively. Among the patients with moderate stereoacuity, 63% improved to “good,” and 37% showed no change. Among the patients with good stereoacuity, 93% remained “good” and 7% deteriorated to “moderate” postoperatively (Fig. 1). In group B, 89% of the patients with poor preoperative stereoacuity showed improvement (44.5% good and 44.5% moderate) and 11% showed no change. Among the patients with moderate stereoacuity, 58% improved to “good,” and 42% showed no change. Eighty nine percent of the patients with good stereoacuity remained “good” postoperatively and 11% deteriorated to moderate postoperatively (Fig. 2). The rates of “fusion” for the Worth 4-dot test (Table 4) were 53.7% in group A and 37.2% in group B preoperatively, and 74.0% in group A and 44.3% in group B at the final visit. Group A showed a significantly higher fusion rate than group B before surgery and at the final visit (p = 0.007 and p < 0.001, respectively). The rate of fusion significantly increased at the final visit compared to the preoperative result in group A (p < 0.001), but it did not increase in group B (p = 0.263). Additionally, we analyzed the association between clinical variables, including the Worth 4-dot test results, and surgical outcome (success vs. recurrent XT) through logistic regression analysis (Table 5). The results showed that younger age at surgery and fusion in the preoperative Worth 4-dot test were associated with postoperative surgical success.

**Table 3:** Titmus stereotest results in groups A and Band clinical variables.

	Group A (n = 177)	Group B (n = 126)	p-value^†^
**Preoperative**			p-value^1^ = 0.991
Good	102 (57.6%)	72 (57.1%)	
Moderate	63 (35.6%)	45 (35.7%)	
Poor	12 (6.8%)	9 (7.1%)	
**At final follow-up**			p-value^1^ = 0.726
Good	138 (78.0%)	94 (74.6%)	
Moderate	37 (20.9%)	31 (24.6%)	
Poor	2 (1.1%)	1 (0.8%)	
	p-value^2^ = 0.000	p-value^2^ = 0.003	

Group A = alignment between 10 prism dioptres (PD) exodeviation and 5 PD esodeviation at the last follow-up; Group B = residual or recurrent exotropia defined as exodeviation > 10 PD at last follow-up.

p-value^1^: Comparison between group A and group B.

p-value^2^: Comparison between pre- and post-operative (at final follow-up) results.

Titmus stereotest grading: good (40–60 arcsec), moderate (80–200), and poor (> 200).

^†^Chi-square test.

**Table 4:** Rates of fusion for Worth 4-dot test in groups A and B.

	Group A	Group B	p-value*
Preoperative	80/149 (53.7%)	45/121 (37.2%)	p-value^1^ = 0.007
At final follow-up	111/150 (74.0%)	51/115 (44.3%)	p-value^1^ = 0.000
	p-value^2^ = 0.000	p-value^2^ = 0.263	

Group A = alignment between 10 prism dioptres (PD) exodeviation and 5 PD esodeviation at the last follow-up; Group B = residual or recurrent exotropia defined as exodeviation > 10 PD at last follow-up.

p-value^1^: Comparison between group A and group B.

p-value^2^: Comparison between pre- and post-operative (at final follow-up) results.

*Chi-square test.

**Table 5:** Logistic regression analysis of surgery success and clinical variables.

Variables	P-value	Odds ratio
Sex	0.137	0.663 (0.383,1.141)
Age at surgery (years)	0.003	1.126 (1.040,1.219)
Preoperative angle of exodeviation (PD) at far	0.266	0.968 (0.915,1.025)
Preoperative angle of exodeviation (PD) at near	0.983	1.000 (0.964,1.038)
Vertical deviation^c^	0.907	1.049 (0.468,2.354)
Oblique muscle dysfunction	0.108	1.894 (0.870,4.125)
Dissociated vertical deviation	0.163	0.164 (0.013,2.075)
Postoperative follow-up period (months)	0.199	0.996 (0.990,1.002)
Titmus stereotest	0.682	0.911 (0.582,1.425)
Worth 4-dot test	0.028	1.825 (1.066,3.125)

## Discussion

Measuring the size of exodeviation angles and the frequency of manifest or tropic phase of exodeviation (‘the fusional control’) has been known as the way to assess the severity of X(T). The fusional control can be judged by home or office control and stereoacuity^5,25^. Previous studies have recognized that loss of control of distance deviation precedes loss of control at near; therefore, assessing the binocular sensory status of the patient at far distance may provide an early measure of the degree of exodeviation control^3,13,25^. O’Neal et al. reported that diminished distance stereoacuity is an objective measure of loss of control in X(T), and surgical correction of exodeviation leads to significant improvement in distance stereoacuity^12^. Conversely, near stereoacuity does not correlate well with fusional control of exodeviation and surgical outcomes^2,11,26–28^. Stathacopoulos et al.^2^ revealed that there was no difference in near stereoacuity between the normal control group and patients with X(T), and Baker and Davies^17^ reported that near stereoacuity showed no correlation with the surgical outcome in 87.1% of the patients with X(T). However, Lee et al. reported that near stereopsis is a useful tool for the assessment of initial sensory status as well as postoperative prognosis, and even in patients with poor preoperative stereoacuity, near stereoacuity, and near sensory fusional status showed postoperative improvements^15^. Similarly, our study indicated that stereopsis assessed with Titmus stereotest at the final visit was significantly more improved than that of the preoperative result in both successful motor outcome and residual or recurrent exotropia groups (p < 0.01).

Several reports demonstrated that diminished distance stereoacuity may not be the most sensitive indicator of X(T) control; rather, central suppression occurs before loss of distance stereoacuity^20,21^ The distance Worth 4-dot test, which evaluates the status of central fusion, had significantly better results for the successful motor outcome group than the recurrent exotropia group preoperatively and postoperatively, in the present study. Moreover, only patients with good motor outcomes had a statistically significant improvement in the Worth 4-dot test after surgery. These results suggest that the preoperative distance Worth 4-dot test may be useful for predicting postoperative motor prognosis. The results of this study were consistent with those of Yilderim et al.^20^, who demonstrated a correlation between the distance alternate-letter suppression test and surgical success.

There were several limitations to the present study. First, it had a retrospective design, and the surgeon selected the surgery to be performed without any standardized criteria, although she had no preference for either the BLR or R&R procedures. However, there was no statistical difference in the results based on surgical procedures between groups A and B. Second, patients with successful motor outcomes (group A) were younger and had smaller exodeviation angles at distance than group B, which may have caused a minor bias. Additionally, whether the improvement in the stereopsis has an effect on the improvement in the post-operative motor success or whether the improvement in the angle of deviation after surgery affects the improvement in the stereopsis is still unclear. Therefore, these results need confirmation in further randomized prospective studies.

In conclusion, Titmus stereoacuity significantly improved after surgery in patients with both successful motor outcomes and recurrent exotropia; however, there was no significant difference between the two groups. The rate of fusion for the distance Worth 4-dot test significantly increased postoperatively in the successful motor outcome group only, and it was significantly higher in the successful motor outcome group than in the recurrent exotropia group pre- and postoperatively. Better central fusion was frequently associated with better surgical success in X(T). Therefore, the distance Worth 4-dot test may be useful for evaluating postoperative prognosis and preoperative sensory status. Further randomized prospective studies, with a large sample of patients with intermittent exotropia, are needed to evaluate the correlation between stereoacuity (near/distance) using various types of stereoacuity tests and the Worth 4-dot test with clinical measures for fusional control.

## Data Availability

All data relevant to the study are included in the article.
